# Characterization of microbial populations in two distinct dairy manure management systems: seasonal effect and implications for pollutant gases emissions

**DOI:** 10.1093/jas/skae316

**Published:** 2024-10-26

**Authors:** Esperanza Fuertes, Ahmad Reza Seradj, Joaquim Balcells, Jordi Maynegre, Gabriel de la Fuente

**Affiliations:** Department of Animal Science, Agrotecnio Center, Universitat Lleida, Alcalde Rovira Roure 191, Lleida 25198, Spain; Department of Animal Science, Agrotecnio Center, Universitat Lleida, Alcalde Rovira Roure 191, Lleida 25198, Spain; Department of Animal Science, Agrotecnio Center, Universitat Lleida, Alcalde Rovira Roure 191, Lleida 25198, Spain; Department of Animal Science, Agrotecnio Center, Universitat Lleida, Alcalde Rovira Roure 191, Lleida 25198, Spain; Department of Animal Science, Agrotecnio Center, Universitat Lleida, Alcalde Rovira Roure 191, Lleida 25198, Spain

**Keywords:** dairy cows, housing systems, manure management, manure microbiota

## Abstract

Following an increase in the demand for dairy products, higher quantities of manure are consequently produced, with the subsequent pollutant gas emission charge associated with its management. The 2 mostly used housing systems in the northeast of Spain, cubicles (CUB) and compost-bedded pack (CBP), entail different manure management techniques; thus, our main objective was to describe the microbiota present in the manure of both systems during 2 distinct climatic situations (winter, mean temperature of 6.2 °C; and summer, mean temperature of 36.4 °C). The secondary aim was to correlate these microbiological profiles with literature findings on the emission of certain well-known pollutant gases from manure. CBP showed to have higher alpha biodiversity as well as presenting a remarkable clustering by season but showed lower network complexity than CUB. Firmicutes/Bacteroidetes ratio was found superior in CUB, which also presented a significantly higher abundance of methanogenic genera belonging to Euryarchaeota phylum, such as *Methanobrevibacter, Methanosaeta* or *Methanosarcina*. On the other hand, CBP manure presented a significant presence of *Corynebacterium, Pseudomonas,* or *Truepera*, among other genera, which activity has been linked to nitrogen (N) transformation pathways in manure. The season also had a relevant role to play in the fluctuation of these populations within each housing system under study. These results show how microbial populations change when manure is differently managed, and how these variations can be related to the synthesis of certain pollutant gases in housing systems.

## Teaser

This study reveals how different manure management systems in dairy farming impact microbial communities, therefore playing an important role in derived pollutant gas emissions.

## Graphical Abstract

**Graphical Abstract:**
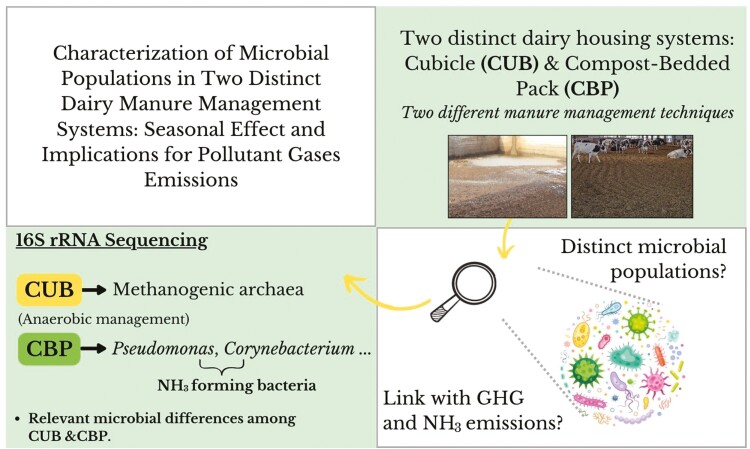

## Introduction

According to the Department of Agriculture, Livestock, Fisheries and Food (DACC, 2023), the Lleida region (northeast of Spain) accounts for the largest dairy farms in the area. It is responsible for over 40% of the bulk milk production in Catalonia, leading it to become the third-largest dairy-producing community in Spain (MAPA, 2023). As a consequence of this great milk production, high amounts of manure are also generated daily.

Manure is mostly composed of organic matter (OM) and minerals that act as a reservoir for a wide range of microbial populations. In turn, these populations are responsible for the emission of relevant amounts of manure-source GHG and NH_3_ (Sanchis et al., 2019; Feng et al., 2023). Previous works have found differences in gaseous emissions coming from distinct dairy housing systems (Owen and Silver, 2015; Poteko et al., 2019; Qu et al., 2021), but there is still a need to give an explanation for such distinctions from a microbiological point of view.

In Spain, specifically within the Catalonia region, 2 primary dairy management systems are identified, which are cubicles (CUB) and compost-bedded packs (CBP). CUB consists of a free-stall system where manure is frequently scraped from the concrete floor of the barn and stored in an open-air storage. On the other hand, CBP involves an open resting area where the bedding material is made up by the own cattle manure, which is daily composted in situ. This procedure enhances aerobic decomposition, improving the aeration and accelerating the decomposition rate, providing benefits for the welfare and health of the animals (Endres and Barberg, 2007; Black et al., 2013). Nevertheless, this aerobic composting process has been found as a trigger for higher NH_3_ volatilization rates in comparison with CUB systems (Balcells et al., 2020).

Identifying manure microbial populations and their interactions would improve the understanding related to changing emissions from dairy barns under distinct manure handling practices. Therefore, the objective of this study is to characterize microbial populations in stored manure from the 2 main dairy housing systems in the northeast of Spain (CUB and CBP), as well as to determine the seasonal effect of winter and summer temperatures on the dynamics of those populations in each housing system.

## Material and Methods

### Barn selection

Measurements were conducted in 6 selected commercial dairy farms in Lleida’s surroundings, northeast Spain. The chosen farms were close to each other to ensure equivalent climatic conditions. Barns 1 to 3 were equipped with a conventional free-stall system based on cubicles (CUB) counting with an automatic circulating manure scraper system. The barn concrete floor was scraped every 2 to 4 h and produced manure was conducted and stored in an outside concrete open-air storage pool. Barns 4 to 6 were provided with a compost-bedded pack (CBP), which was tilled twice daily at the same hour and at about 25 cm depth. No bedding material was used apart from the own cow dung, with seldom straw incorporation during the winter season to reduce pack humidity. Barn selection aimed to achieve both a diverse representation of the distinct dairy management systems as well as a consistent level of uniformity among the barns included in the study. Specific characteristics of each farm participating in this study can be found in Supplementary Table 1.

### Animal management

Holstein Friesian cows were raised on all farms, with a mean lactation number of 2.2 for CBP and 2.3 for CUB. The mean number of animals ranged from 230 to 273 for CBP and from 231 to 220 for the CUB system between both seasons under study. The animals underwent artificial insemination around 157 d after calving and were dried off 63 d prior to the next calving, resulting in approximately 83.6% of the year spent in milk production. Regarding the diet, cows were provided with TMR balanced in accordance with the guidelines of the Agricultural and Food Research Council (1993). The diets consisted of a minimum of 45% forage including corn and alfalfa silage, to support a daily milk production of 30 to 35 kg/cow. Expressed per dry matter (DM), crude protein ranged between 16% and 17%. The greatest possible similarity for both ingredients and proportions between the diets administered in each farm was sought. More details referring to the cows’ performance can be found in Supplementary Table 2.

### Sampling collection and seasonal differentiation

Manure samples (≈ 200 g sample^−1^, ≈ 40 samples per housing system) were taken from both housing systems in 2 different seasons: winter (January or February) and summer (July or August). Within the CBP system, samples were taken from the central area (at least 4 m lengths from the barn wall) of the compost bed between 0 and 30 cm deep by a steel drill designed for soil sampling (7 cm in diameter × 100 cm long, Eijkelkamp, Nijverheidsstraat, Giesbeek, The Netherlands). Samples from the CUB system were taken from the storage pool at 0.5 m length from the pool wall through a volumetric PVC probe at a similar deepness than in CBP. Crust formation upon the slurry pit was mostly seen during winter samplings, mainly in barns 1 and 3; collecting material from that layer was avoided as much as possible. By performing 2 sampling days per season, selecting samples from at least 2 distinct areas per day, between 13 and 14 samples were taken per farm, with a final result of 82 manure samples to be analyzed.

Throughout the experimental period, temperature and wind speed data were collected from the nearest climatic control stations within a distance of less than 20 km [Torres de Segre (41º51ʹ90.9″N 0º55ʹ31.4″E), close to barn 1 and 5, El Poal (41°40ʹ15.0″N 0°52ʹ37.7″E), close to barns 2 and 3, and Torres de Segre (41°47ʹ08.1″N 0°49ʹ40.5″E), close to barns 5 and 6]. Temperatures ranged from 31 °C to 37.5 °C in summer, and from 3.7 °C to 7.1 °C in winter. Precipitations were inexistent during the summer period, while the mean value for the winter sampling was also very low (1.94 mm/m^2^). Unfortunately, the inner pack temperature was not recorded. Samples were rapidly frozen at −30 °C after collection for further analysis.

### Chemical analysis

Manure DM was obtained in thawed samples by a forced-air oven at 105 °C for 24 h. Total N and NH_3_-N contents were analyzed following the Kjeldhal method and direct distillation with Na_2_B_4_O_7_ (AOAC, 2010). Carbon (C) was analyzed by combustion analysis (Truspec CHN, Leco). Volatile solids (VS), which correspond to the OM fraction present in manure, were estimated through ignition at 550 °C for 3 h using a Leco model (LECO Corp., St. Joseph’s, MI). pH was determined with a pH meter (Crison micropH 2000).

### DNA extraction and sequencing

DNA extraction and sequencing were carried out on freeze-dried manure samples by Microomics Systems, S.L. (Barcelona, Spain). Following the manufacturer’s instructions, the DNeasy PowerLyzer PowerSoil Kit (Qiagen, Hilden, Germany) was used to extract microbial genomic DNA. A mock community was included as a positive control for library preparation (Zymobiomics Microbial Community DNA, ZymoResearch, Irvine, CA, USA). Samples were amplified using primers 341F and 805R, which target the V3–V4 region of the bacterial and archaeal 16S rRNA:

## Forward

5ʹ - TCGTCGGCAGCGTCAGATGTGTATAAGAGACAGCCTACGGGNGGCWGCAG.

## Reverse

5ʹ—GTCTCGTGGGCTCGGAGATGTGTATAAGAGACAGGACTACHVGGGTATCTAATCC.

PCR was performed in 10 μL final volume with 0.2 μM primer concentration. The PCR included: 3 min at 95 °C (initial denaturation) followed by 25 cycles of 30 s at 95 °C, 30 s at 55 °C and 30 s at 72 °C, and a final elongation step of 5 min at 72 °C. PCR products were purified using AMPure XP beads (Beckman Coulter, Nyon, Switzerland) with a 0.9 × ratio according to the manufacturer’s instructions.

Sequencing was performed on an Illumina MiSeq 2 × 300 platform. Quality control filtering and OTU binning of the resulting sequences were executed using DADA2 software (Callahan et al., 2016). Taxonomic assignment of phylotypes was performed using a Bayesian Classifier trained with Silva database version 132 (99% OTYs full-length sequences; Wang et al., 2007). Bioinformatic procedures were carried out by Microomics Systems, S.L. Bioinformatic analysis using R software version 4.3.2 was performed on the resulting data.

### Statistical analysis

Alpha diversity comparisons were performed using the Kruskal–Wallis non-parametric test in order to measure the variability of species within a sample and were assessed by Shannon, Simpson, and Evenness indexes and microbial Richness.

To measure differences in microbiome composition between samples, a beta diversity distance matrix was applied through a partial least square discriminant analysis (PLS-DA) based on CLR (mixOmics package). Both Adonis (permutational multivariate analysis of variance) and Anosim tests were applied to determine the differences in the community structure between groups at a significance of *P *< 0.05 after 10,000 random permutations (Vegan package). To determine which genera abundance were responsible for the differences between groups, an ANOVA-like differential expression analysis (ALDEx) was performed (Aldex2 package).

To describe the interactions in the microbial community within both systems and both seasons, we performed a network analysis through the Sparse Correlations for Compositional data technique (SpiecEasi package). Microbial networks were graphically represented (igraph package) and their complexity was explained as the number of nodes (genera), number of edges (significant positive or negative correlations), node degree (number of connections that any node establishes with other nodes), and betweenness centrality (a measure of centrality in a graph based on shortest paths). Connexions were considered at R > 0.6 and *P *< 0.05. Parameters such as chemical and environmental characteristics were analyzed through the Mixed Procedure in SAS (version 9.4; SAS Institute Inc., Cary, NC, USA) in a 2 × 2 model to determine the effect of season, housing system, and their interaction, declaring significant effects at *P* < 0.05.

## Results

### Herd performance and environmental variables

None of the parameters related to herd performance presented differences either with respect to the housing system or with the season. Despite being true that there was a great variation in the number of animals among farms (SEM: 50.9), this is due to the fact that farms 2, 3, 5, and 6 had more than one identical pen, resulting in a higher absolute number of cows. Results regarding mean lactations, parturition interval, DMI, or milk yield were similar to those registered by Balcells et al. (2020) and within the intervals proposed by FEFRIC (2021).

Climatic conditions during the sampling differed between seasons, where temperature (36.4 °C in summer vs. 6.2 °C in winter, *P *< 0.01) showed significant variations. The climatic control stations under study did not present relevant temperature record differences.

### Manure chemical characteristics

Manure chemical characteristics are presented in Table 1. In manure samples, DM (g 100g FM^−1^) was higher in CBP than in CUB (38.0 vs. 12.85; *P *< 0.001) as the composting process performed in CBP systems leads to drier manure. The concentration of N (g kg DM^−1^) in CUB barns was significantly higher than that in CBP barns (35.8 vs. 29.5; *P *< 0.001), as well as presenting slightly higher concentrations during the coldest season for both systems. NH_3_-N also presented significantly higher concentrations in CUB manure with respect CBP system.

**Table 1.** Chemical characteristics of manure from both cubicles (CUB) and compost-bedded pack (CBP) during summer and winter periods

Item	CBP		CUB		SEM	*P* value		
	Summer	Winter	Summer	Winter		HS^s^	Se	HS × Se
DM, g 100 g FM^−1^	37.3	38.7	12.6	13.1	1.09	<0.001	0.66	0.81
VS, g 100 g DM^−1^	75.8	79.2	71.8	72.3	0.71	<0.001	0.17	0.32
N, g kg DM^−1^	28.6	30.5	35.1	36.5	0.05	<0.001	0.02	0.77
NH_3_−N, g kg DM^−1^	7.57	6.45	20.15	18.01	1.35	<0.001	0.55	0.85
Ratio C:N	11.8	14.6	10.3	9.62	0.47	<0.001	0.29	0.08
pH	8.22	8.16	7.58	7.51	0.05	<0.001	0.57	0.93

HS, Housing System; Se, Season.

C:N ratio, describing the present amount of C in relation to the amount of N, was found significantly superior in CBP systems; within this system, the winter period presented a higher C:N ratio. VS were also superior in CBP, with both systems presenting higher concentrations during winter. Regarding pH values, more alkaline results were found in CBP systems than in CUB (8.19 vs. 7.54), with no seasonal effect.

### Microbial community diversity

After sequencing and normalization, a mean number of 25,050 sequences per sample was obtained, generating a total of 2,029,053 sequences from the 82 samples. A total of 766 OTUs were found. The unclassified mean rate of OTUs at the general level was 0.04 %.

Alpha biodiversity was assessed by Shannon, Simpson, and Evenness indices and microbial Richness (values presented in Table 2). CBP showed to have higher microbial biodiversity, in terms of Shannon and Simpson indexes, for both seasonal periods. CBP system presented as well higher number of OTUs based on the Richness parameter for both seasons, with no statistical differences between them.

**Table 2.** Manure microbial alpha biodiversity from both cubicles (CUB) and compost-bedded pack (CBP) during summer and winter periods

Item	CBP		CUB		SEM	*P* value		
	Summer	Winter	Summer	Winter		HS	Se	HS × Se
Shannon	4.54	4.50	4.31	4.07	0.02	<0.001	0.006	0.06
Simpson	0.98	0.97	0.97	0.96	0.001	<0.001	<0.001	0.008
Richness	204.5	209.6	161.4	150.1	3.68	<0.001	0.67	0.27
Evenness	0.85	0.84	0.85	0.82	0.002	0.004	<0.001	0.04

HS, Housing System; Se, Season.

**Figure 1.**
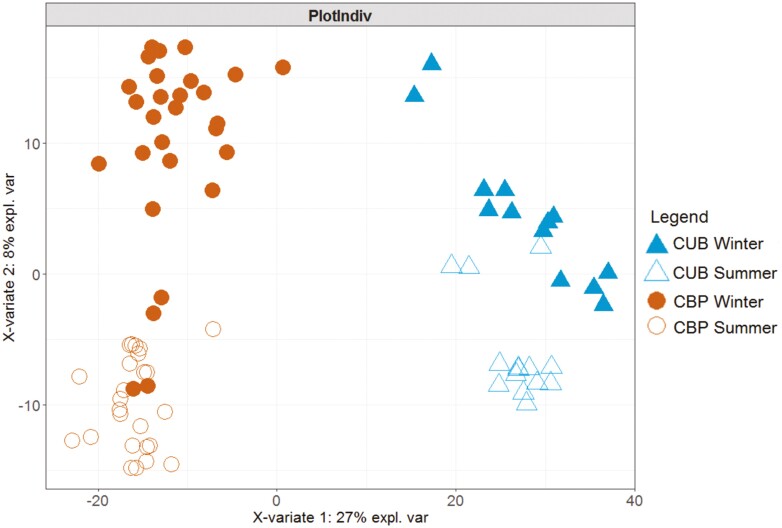
Graphical representation of partial least squares-discriminant analysis (PLS-DA) on bacterial and archaeal OTUs present in manure from cubicles (CUB) and compost-bedded pack (CBP) manure during summer (SUM) and winter (WIN).

Partial least squares-discriminant analysis (PLS-DA) was conducted to analyze microbial beta biodiversity in the study, graphically represented in Figure 1. Samples were clearly clustered by system, and within each system, it was found separation between seasons, which was remarkable in the case of CBP. Adonis test highlighted significant differences between systems (*P* < 0.001) and periods (*P *< 0.001). Finally, the Anosim test indicated a larger between-groups than within-groups variability, strengthening the hypothesis of both season and housing impact on the manure microbial population structure.

**Figure 2.**
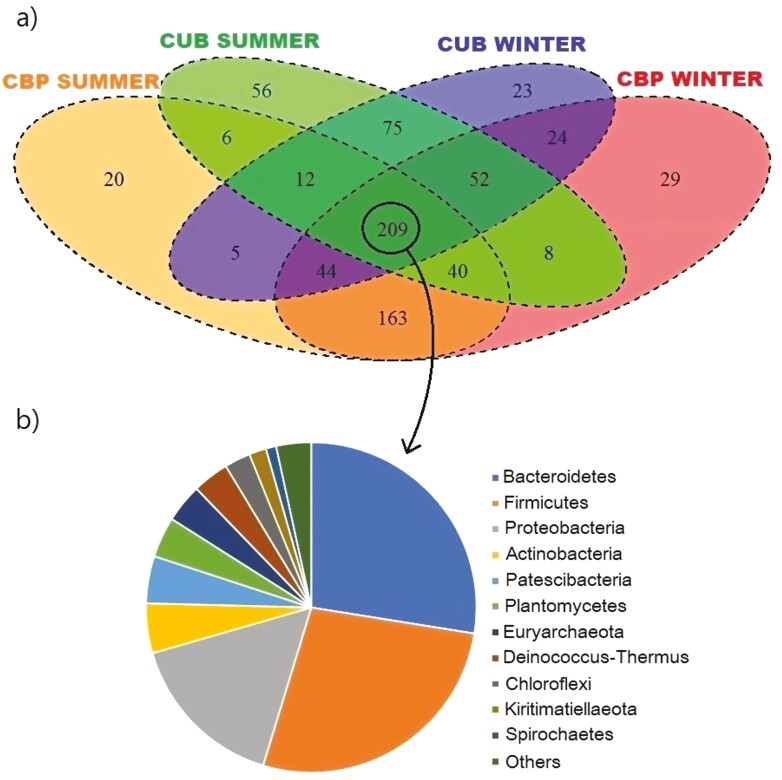
(A) Venn diagram showing the numbers of bacterial and archaeal taxa that are shared or unshared by groups from cubicles (CUB) and compost-bedded pack (CBP) manure during summer and winter; (B) Shared sequences across all samples (core population).

To determine the proportion of shared and unshared OTUs, a Venn diagram was performed (Figure 2). Even though a core microbiota exists across groups, there were unique taxa that changed within the system and season. Inside those shared populations among the 4 groups, the predominant phyla were Bacteroidetes (27%), Firmicutes (27%), Proteobacteria (16%), Actinobacteria (5%), and Patescibacteria (5%).

**Figure 3.**
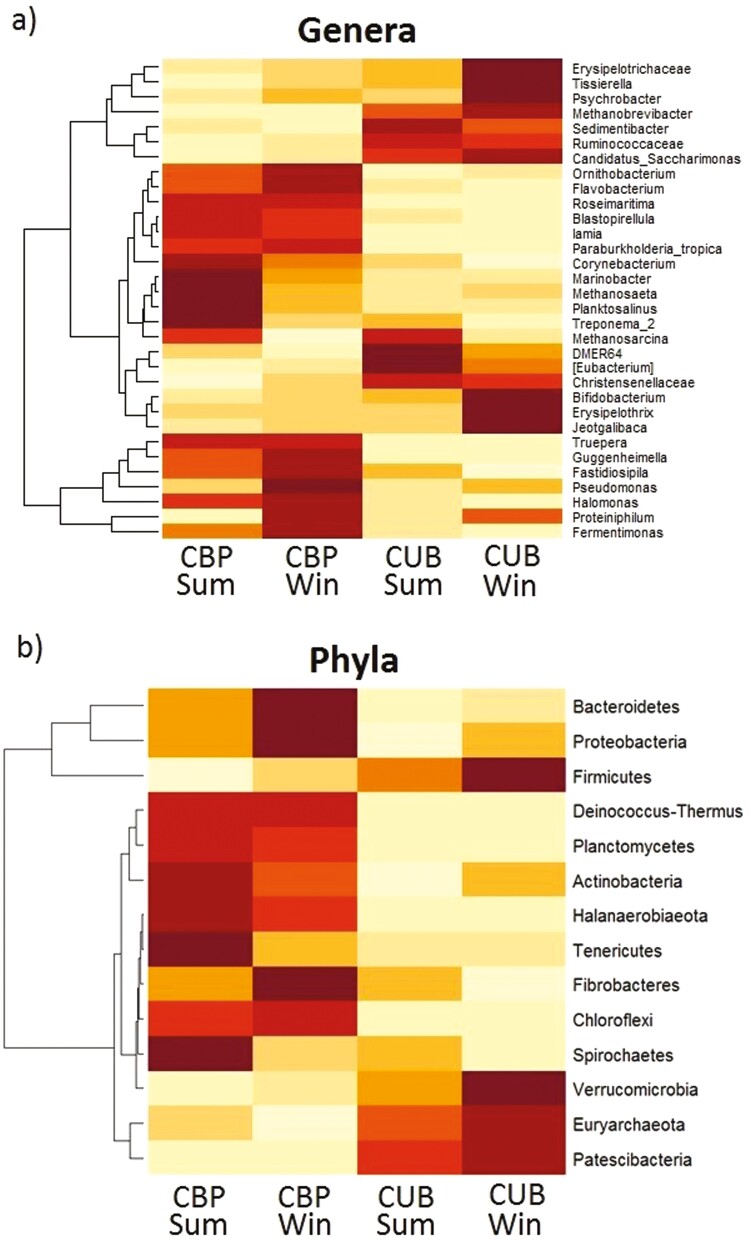
Microbial community diversity expressed on (A) genera and (B) phyla from cubicles (CUB) and compost-bedded pack (CBP) manure during summer (SUM) and winter (WIN). Darker colors signify higher abundance for that genera or phyla among the 4 groups.

The microbial diversity assessment using phylotype taxonomy resulted in a total of 766 OTUs, from which the 60 most abundant genera were selected, all of them with a relative abundance above 0.25%. A heat map of the top genera present in manure from both management systems in both seasons under study is shown in Figure 3A. Within them, the most abundant genera in CBP system were *Fermentimonas* (9.3%), *Proteiniphilum* (9.1%), *Halomonas* (9.0%) and *Truepera* (6.3%), while in CUB were *Proteiniphilum* (9.9%), *Methanobrevibacter* (9.0%), *Candidatus Saccharimonas* (6.6%), and *Fermentimonas* (5.6%). Those 60 most abundant genera belong to 14 different phyla, which are also represented in Figure 3B. Dendrograms on the left of each heat map show the clustering among genera and phyla, showing differentiation in abundance between groups, with a clear separation between housing systems.

**Figure 4.**
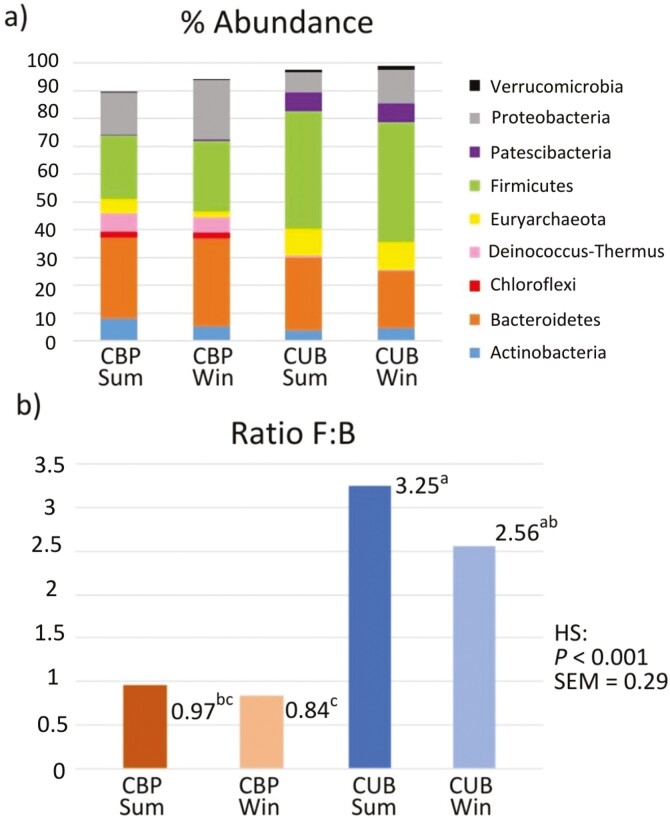
(A) Phyla distribution of most abundant genera (relative abundance above 0.25%) (B) Ratio Firmicutes/Bacteroidetes (F/B) from cubicles (CUB) and compost-bedded pack (CBP) manure during summer (SUM) and winter (WIN).

Moreover, in Figure 4A predominant phyla within the 0.25% of relative abundance are graphically displayed for the 4 groups. Again, differentiation in proportions among housing systems was greater than those occasioned by the seasonal variation. The most abundant phyla in CBP were Bacteroidetes (30.8%), Firmicutes (23.7%), Proteobacteria (19.7%), and Actinobacteria (6.5), while in CUB were Firmicutes (42.7%), Bacteroidetes (23.0%), Proteobacteria (10.1%), and Euryarchaeota (9.8%). Ratio Firmicutes:Bacteroidetes are also presented in Figure 4B, being only affected by the housing system and showing higher ratios for both seasons of the CUB system.

### Microbial networks

**Figure 5.**
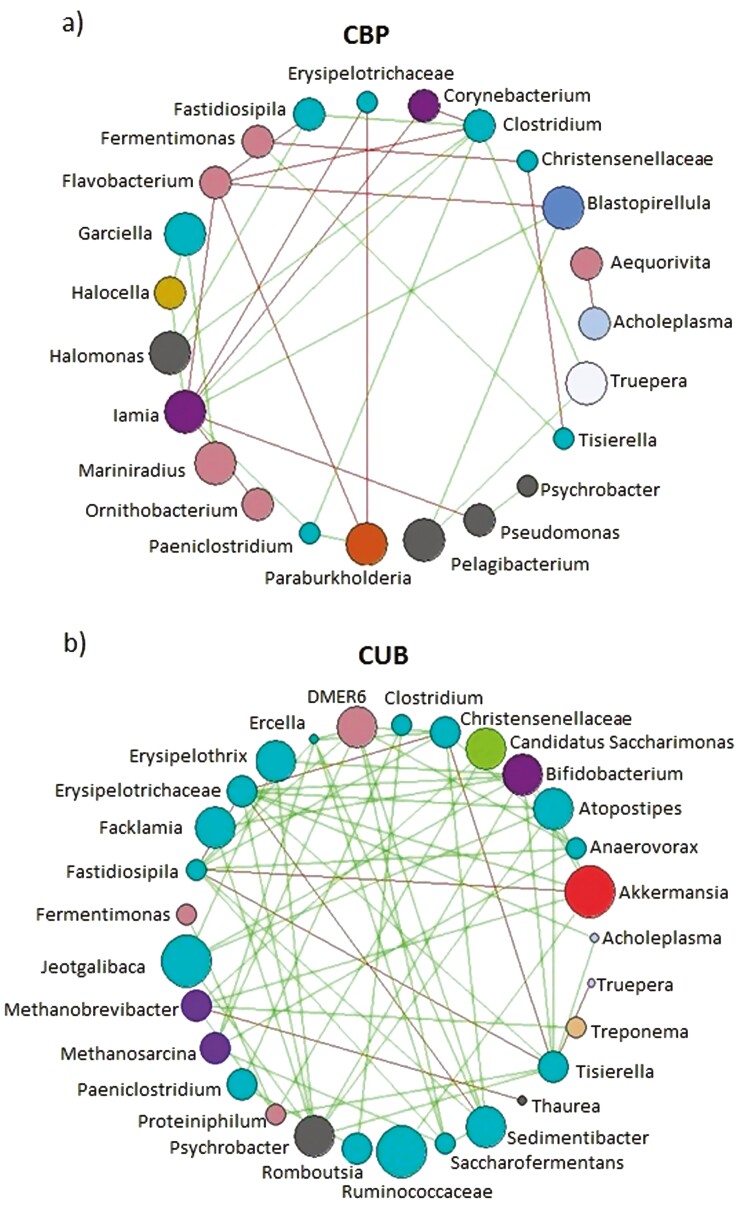
(A) Networks on compost-bedded pack (CBP) system genera; (B) Networks on cubicles (CUB) system genera. Correlations were generated establishing significant correlations at R *> *0.60 and *P* *< *0.05. Edges indicate positive and negative correlations. Identically colored nodes share the same phylum.

Microbial networks were performed to test microbial interactions by co-occurrence patterns (Figure 5). The degree of interaction was exposed as the number of taxa (nodes) that establish significant interactions (edges) with other taxa.

CBP system presented a lower microbial network complexity with respect CUB system in terms of nodes (23 vs. 29), but the greatest difference was seen regarding edges, where CUB doubled CBP interactions (63 vs. 30). Negative and positive correlations were quite balanced in CBP, while CUB system just presented 8 negative edges out of the 63 correlations found. More than 55% of genera found in CUB systems belong to Firmicutes phylum (turquoise-colored nodes).

Network metrics established per season on each housing system are presented in Table 3. Network dynamics varied differently between seasons on each system; while the winter period seemed to stimulate more connections on CUB, the opposite was found in CBP systems. CUB manure presented more connected networks than CBP in both the summer and winter seasons, with a greater number of edges, especially in winter (261). The genera with the highest number of associations in CUB were *Tissierella, Fastidiosipila,* and *Erysipelotrichaceae*, with 9 connections, while CBP’s most connected node was *Iamia*, also with 9 connections.

**Table 3.** Networks metrics described in cubicles (CUB) and compost-bedded pack (CBP) manure during summer and winter

Parameter	CBP summer	CBP winter	CUB summer	CUB winter
Number of nodes	38	40	42	46
Number of edges	92	70	125	261
Abundance connected networks	0.87	0.88	0.94	0.97
Average node degree	4.84	3.50	5.95	11.34
Average betweenness	6.81	3.87	6.90	7.36

Betweenness indicates which nodes are important in the flow of the network, making use of the shortest paths. Therefore, the higher the betweenness, the more the presence of important nodes working on the efficient flow of goods in the network. In this way, during the summer period, betweenness was almost equal between both systems, but while it decreased significantly in winter on the CBP system (3.87), CUB had the highest betweenness value in that season (7.36), meaning that this was the system and the period with better network efficiency.

## Discussion

Manure can chemically be considered as a complex of OM containing minor minerals. As soon as the manure is excreted, it undergoes a series of reactions such as decomposition, hydrolysis, nitrification, denitrification, or fermentation, among others, from which certain GHG as well as NH_3_ can be produced (Asselin-Balençon et al., 2014; Pratt et al., 2015). Since aerobic decomposition requires oxygen as an electron acceptor, better aeration accelerates the decomposition rate, which is what the tilling process promotes in CBP systems. However, decomposition can also take place under anaerobic conditions, such as those found more likely on CUB systems, where manure is stored in open-air storages in liquid form.

Most of the relevant literature has focused on the identification of microbial populations at the gut level due to the relevance of enteric emissions. Nevertheless, it is needed to expand the existing information in the field of microbial populations present in dairy cattle manure due to its specific contribution to the global pollutant gas emissions, paying specific attention to the different housing systems.

We hypothesized that manure microbiome changes, not only when manure management differs, but also under different climatic temperatures. These changes, in turn, could be responsible for the pollutant gas emission differentiation between housing systems and seasons described in the literature (Galama et al., 2015; van Dooren et al., 2016). Thus, understanding the effect that management practices and environmental conditions have on manure microbial populations might contribute to a broader comprehension of such emissions.

### Animal management

Given the great importance that the manure to be studied must be as compositionally identical as possible between farms, diets and management conditions used in the 6 barns participating in the study were highly similar. Moreover, the impact of farm location was analyzed in both alpha and beta diversity, and no significant differences were found among them (*P* > 0.05). Herd performance did not present differences among the barns under study.

### Chemical composition of manure

Temperature variations and manure management techniques can influence manure composition (Atzori et al., 2016). As diet differences were minimal among barns or housing systems, we attribute changes in manure’s chemical characteristics to handling and seasonal variations.

In manure samples, DM was higher in CBP than in CUB (38.0 vs. 12.85; *P* < 0.001), reflecting the drier manure resulting from the composting process in CBP systems. VS levels were within reported ranges (Petersen, 2018), with higher winter quantities consistent with the literature (Grant et al., 2015; Cárdenas et al., 2021), possibly due to accelerated VS degradation in warmer temperatures (Sawamoto et al., 2016). Lower VS in CUB barns may indicate more CH_4_ conversion in the anaerobic conditions of liquid CUB manure storage (IPCC, 2006; de Boer, 2014).

Nitrogen (N) concentration was significantly higher in CUB barns compared to CBP barns (35.8 vs. 29.5; *P* < 0.001), with higher concentrations in colder seasons for both systems. NH_3_-N was also higher in CUB (*P* < 0.01), aligning with Balcells et al. (2020) and Fuertes et al. (2021), which found greater NH_3_ volatilization in CBP, resulting in lower final N and NH_3_-N levels in CBP manure.

The C:N ratio impacts manure degradation, with optimal ratios around 30, though dairy manure typically ranges from 15 to 19 (Black et al., 2013; Leso et al., 2020). Ratios below 15 can hinder composting and increase N release (Brust, 2019). Adding bedding material raises the C:N ratio and reduces moisture, benefiting both composting and animal health. Our results showed C:N ratios in CBP systems were higher than in CUB systems but did not reach optimal values (11.8 in summer and 14.6 in winter), as liquid manure storage typically results in lower ratios (Powel-Smith, 2023). The higher winter ratio in CBP is due to added straw, increasing C availability.

pH showed no seasonal effect, but the housing system significantly impacted pH, with CBP manure being more alkaline than CUB (8.19 vs. 7.22; *P* < 0.001). Crust formation contributes to lower pH in CUB (Blanes-Vidal et al., 2008), while in CBP, urea hydrolysis and CO_2_ evaporation during aeration raise the pH (Montes et al., 2009; Li et al., 2012).

### Housing system effect on microbial community

The PLS-DA plot (Figure 1) clearly shows that CBP microbial samples clustered separately from CUB samples, a result confirmed statistically by the Adonis test. Figure 2 further illustrates the separation of shared and unshared OTUs between housing systems and seasons, along with the phyla composition of shared OTUs among the 4 groups.

To explore the main taxa contributing to these differences, we generated a heat map of the most abundant genera (relative abundance >0.25%) and phyla (Figure 3). Both genera and phyla were primarily clustered by housing system, with minimal seasonal influence.

In CBP, the most abundant genera, *Fermentimonas* and *Proteiniphilum* (both from the Bacteroidetes phylum), were consistent with previous findings in dairy compost bedding (Ren et al., 2016; Sun et al., 2020). While *Proteiniphilum* was also dominant in CUB, the next most abundant genera differed between the 2 systems. *Halomonas* and *Truepera* were more prevalent in CBP, whereas CUB had higher levels of *Methanobrevibacter* and *Candidatus Saccharimonas*. *Halomonas* (Proteobacteria phylum) is common in the thermophilic phase of dairy manure composting (Zhong et al., 2020), while *Truepera* has been observed in mature compost (Wang et al., 2022). The presence of *Candidatus Saccharimonas* in CUB, linked to dairy cows with laminitis (Guo et al., 2021), aligns with studies reporting reduced lameness in CBP systems (Astiz et al., 2014; Burgstaller et al., 2016).

Regarding phyla, Firmicutes and Bacteroidetes were the most abundant, consistent with microbial environments of the gut (Gyawali et al., 2021). Bacteroidetes dominated in CBP (*P* < 0.001), while Firmicutes were more abundant in CUB (*P* = 0.001), resulting in higher F:B ratios for CUB (Figure 4). Changes in manure management may have influenced these ratios despite uniform dietary conditions across farms. This is supported by Pandey et al. (2018), who found Bacteroidetes dominant in solid manure, and Lin et al. (2016), who identified Proteobacteria and Bacteroidetes as key in swine manure composting, which is consistent with our CBP findings. Strom et al. (2022) observed Firmicutes as dominant in swine slurry, resembling CUB conditions. Specific Firmicutes like *Anaerovorax* found only in CUB, have been previously linked to un-composted cow dung (Girija et al., 2013). Actinobacteria, significantly higher in CBP (*P* < 0.001), is known for its role in OM degradation during composting (Ventura et al., 2007; Sundberg et al., 2013).

The higher presence of Euryarchaeota in CUB systems (*P* = 0.007) is significant for its potential role in CH_4_ emissions from this type of manure management. However, this study did not measure gaseous emissions or microbial activity directly. Instead, it focused on describing the relative abundance of microbial populations in each system to link these findings with existing literature on pollutant emissions. As such, the results were interpreted within the limitations of the available data.

In Figure 5 a deeper analysis of microbial interactions inside both housing systems is shown, where CBP presented a lower microbial network complexity and connection between genera in comparison with CUB. This could mean that the mainly anaerobic environment generated on the CUB storage pool, which is minimally altered, allows more associations (especially positive) among certain microbial communities. As CBP manure is constantly altered, mixing anaerobic conditions on the deeper layers that are as well altered daily by the passage of the rotary hallow, could make more difficult the association between manure microbiota.

### Seasonal effect on microbial community

Temperature not only determines the rate of metabolic activities but it is also the major selective factor for microbial populations. That is why a comparison between the 2 most extreme environmental situations (summer and winter) was performed to seek microbial differences within the same housing system.

Concerning alpha biodiversity, season showed significantly higher values in the summer period for Shannon, Simpson, and Eveness indexes (*P *< 0.05). Those results indicate that warmer temperatures favored higher biodiversity upon manure samples in both housing systems. Figure 1 presents a clear separation among seasons for CBP. Moreover, higher homogeneity between OTUs was found in summer with respect to winter for both housing systems, but this effect was especially remarkable in CUB. This would agree with Resende et al. (2016), who found slight variability during the summer months.

Certain differences could be also appreciated between seasons with respect to genera and phyla composition. Various genera belonging to the Firmicutes group, such as *Tisierella*, *Jeotgalibaca,* or *Erysipelothrix* showed a significant rise in winter with respect to their summer concentrations (*P* < 0.05), with greater differences seen in the CUB system (Figure 3). In the CBP system, the Bacteroidetes phylum also experienced a rise in winter mainly due to a relevant growth in *Proteiniphilum* with respect to its summer concentrations (*P* < 0.05). This led to a rise of 35% in Bacteroidetes in winter, agreeing with the increment of this phylum described by Resende et al. (2016) during the coldest months of the year.

Proteobacteria phylum also presented an important rise in winter for both housing systems due to the significant elevations of certain genera such as *Pseudomonas*, *Psychrobacter,* and *Paracoccus* (*P* < 0.05). For the Euryarchaeota phylum, behaviors did vary between systems. The *Methanosarcina* population increased in summer for both CBP and CUB (*P *= 0.02). *Methanobrevibacter*, especially abundant in CUB, showed a trend towards higher concentrations in winter for both systems (*P* = 0.07), while *Methanosaeta* increased significantly in summer on the CBP system.

The population rise of certain of these genera may result strange as warm temperatures stimulate bacterial growth, reaching its optimal level at 25 to 30 °C, while coldest ones delay it (Pietikäinen et al., 2005). Nevertheless, it must be taken into account that the inner temperature of both the compost bed and the storage pool is not equal to environmental or superficial temperature, as microbial activities raise manure temperature making it biologically active. As it is, the composting process could lead the inner manure layers to reach temperatures such as 65 °C, temperatures that are easier to reach during warmer months (Leso et al., 2020). Those high temperatures enable the CBP system to improve udder health by reducing bacterial counts. Therefore, maybe that increment in the pack temperature would lead to a reduction in certain phyla present in manure that appears to be higher during colder months.

### Methane-related microbiota

*Methanobrevibacter* (Euryarchaeota phylum) was dominant in the CUB system for both winter and summer periods, ranging from 8.6% to 9.4% of relative abundance, respectively. It is important to mention that this genus is the most described methanogenic archaea regarding emissions of enteric origin in ruminants (Chaucheyras-Durand & Ossa, 2014; de la Fuente et al., 2019), a fact that could explain its high presence in manure. Meanwhile, its abundance in CBP barns ranged from 1.5% in winter to 2% in summer. This fact would agree with the higher CH_4_ emissions from liquid storage systems described in the literature compared with composted manure (Le Riche et al., 2016; van Dooren et al., 2016).

The relevant growth of *Methanobrevibacter* in CUB systems during winter could be interpreted with the greater crust formation over the storage pool during cold months, which was already highlighted by (Husted, 1994), that could potentiate the anaerobic ambient of the manure enhancing the methanogenic processes. Nevertheless, crust formation has been appreciated as a potential mitigator for CH_4_ release from manure not only because of constituting a physical barrier but also because of the methanotrophic activity found in slurry crusts (Petersen et al., 2005; Guerrero-Cruz et al., 2021). In this sense, certain aerobic CH_4_-oxidizing bacteria such as Verrucomicrobia phylum were found mostly in the CUB system with winter abundance doubling summer values, as the crust was mostly seen in winter. Proteobacteria, also described as an aerobic methanotrophic phylum (Stein et al., 2012), was nonetheless found superior in CBP samples (19.7% vs. 10%), meaning CH_4_-oxidizing activity would be happening also upon CBP manure. As we did not measure the activity of any microorganism described in this work but focused on their abundance, wider work on this topic would be interesting and useful for a better understanding.

The anaerobic environment provided by the CUB storage pool allows methanogenic archaea development; such a fact may explain the greater abundance of Euryarchaeota in the CUB system in both seasons compared with the aerated composted bed in CBP. Nevertheless, Euryarchaeota presence in CBP samples suggests that methanogenic activity may be happening despite the tilling process, probably in the deeper layers of manure. This would agree with existing literature describing CH_4_ emissions from CBP (van Dooren et al., 2016; Fuertes et al., 2023). The *Methanosaeta* genus, also relevant among methanogenic archaea, increased in manure in both CBP and CUB during the summer period, with 2.4% and 1.8% abundance, respectively. *Methanocorpusculum* population, despite not being part of the 60 most abundant genera, was only found in CUB samples; this genus has been described as the most present methanogen in liquid manures (Duan et al., 2014; Habtewold et al., 2017) such as those found in slurry pits storages from CUB systems.

Apart from methanogenic archaea’s higher presence in CUB samples, the bacterial genus *Proteiniphilum* was also found to be more abundant in CUB samples with respect to CBP for both summer and winter periods. This genus has been considered a promotor of methanogenic activity (Pietikäinen et al., 2005), so its higher presence in CUB samples, as well as its positive correlation with Methanobrevibacter (Figure B agrees with the previously stated higher CH_4_ emissions from slurry storages found in the literature.

### Ammonia-related microbiota

Bacterial ureases, present in feces, enable NH_3_ volatilization through urea hydrolyzation when both droppings are mixed (Stewart et al., 2004). Certain bacteria have been found to involve ureolytic activity, such as *Corynebacterium* and *Pseudomonas* (Kigigha et al., 2012; Alizadeh et al., 2017). The latter genus plays also a role in ammonifying processes, degrading organic N into ammonium (NH_4_^+^), being found as one of the only Gram-negative hyper-ammonia-producing bacteria (HAP) in pig slurry (Whitehead and Cotta, 2004; Stremińska et al., 2012). These 2 genera were more than 2 and 3 times higher in CBP than in the CUB system respectively, with significant *P* values. Both systems presented higher concentrations during summer (*P* = 0.001 for both genera).

*Truepera*, which belongs to the Deinococcus-Thermus phylum, was found in the present study as one of the most abundant genus on CBP (6.3%), and its denitrifying activity has been demonstrated in several studies (Zhang et al., 2020; Zhou et al., 2022), therefore taking an important role in N cycle happening upon manure. The same characteristics were found for *Halomonas*, which had a presence of 9% on CBP samples, during the maturation phase of composting (González-Domenech et al., 2010). Genus *Nitrosomonas*, despite not being found among the 0.25% most abundant bacteria, was completely absent in the CUB system while being present in every CBP sample. Zhong et al., (2020) related this genus as one of the most important bacteria involved in the NH_3_ oxidation occurring during the maturation phase of composting; in this way, N losses through the activity of ammonia-oxidizing bacteria should not be neglected in composted dairy manure.

NH_3_ volatilization from manure is known to be enhanced when pH and temperatures rise (Atzori et al., 2016). CBP presented the lower N and NH_3_-N content, as well as the highest pH values (especially in summer), which means better conditions for NH_3_ emission from manure than in CUB systems given; these results highlight the presence of urease-producing bacteria found in CBP systems that are allowing such fast release of NH_3_ from manure.

Regarding the production of N_2_O, a very pollutant GHG that can be also synthesized from manure and which production is closely related to NH_3_ fluctuations (Bhatia et al., 2013), *Flavobacterium*, which is graphically presented with a higher abundance in CBP samples in Figure 3 (*P *< 0.05) was found to be involved in composting denitrification where N_2_O is produced as an intermediate (Chadwick et al., 2011). *Truepera* and *Halomonas,* which were previously mentioned to have a significant presence in CBP samples, have also been related to the denitrification process. Thus, the mixture of aerobic and anaerobic ambient generated in CBP manure could favor the formation of N_2_O as a product of incomplete reactions (Waldrip et al., 2016).

## Conclusions

The distinct microbial communities in CBP and CUB samples might be attributed to the varying effects of the anaerobic process in the CUB environment and the tilling process in CBP. What could be seen as minor variations in manure management do indeed entail a different pattern of microbial communities, as populations varied remarkably among systems. Inside each of them, seasonal variations did affect concentrations of these populations differently. Deeper research has to be conducted to seek quantitative relevance on manure microbial populations in each of the housing systems under study.

## Supplementary Material

skae316_suppl_Supplementary_Table_S1

skae316_suppl_Supplementary_Table_S2

## Abbreviations

CBP: compost-bedded pack system;
CH4: methane
CLR: centered log ratio
CUB: cubicle system
GHG: greenhouse gas;
N: nitrogen;
NH3: ammonia;
N2O: nitrous oxide
OUT: operational taxonomic unit
PLS-DA: partial least squares-discriminant analysis
SUM: summer season
WIN: winter season
