# Supplementary material for: Characterization of microbial populations in two distinct dairy manure management systems: seasonal effect and implications for pollutant gases emissions
Source: J Anim Sci. 2024 Oct 26;102:skae316. doi: 10.1093/jas/skae316 (PMC11641847; doi:10.1093/jas/skae316)
Supplement: skae316_suppl_Supplementary_Table_S1 [file skae316_suppl_supplementary_table_s1.docx]

**Supplementary Material Table 1.** Building characteristics, floor type and manure-handing system in the selected cubicles or compost-bedded pack farms.

| Barn  nº | Barn type | Manure handing system | Equal pens per barn | Manure surface exposed to air (m^2^) | Space allowance (m^2^ barn cow^-1^) |
| --- | --- | --- | --- | --- | --- |
| 1 | CUB | Solid concrete floor, automatic circulating scraper (every 3 hours), lagoon emptying every 3-4 month | 1 | 739.5 | 6.69 |
| 2 | CUB | Solid concrete floor, automatic circulating scraper (every 3 hours), lagoon emptying every 3-4 month | 2 | 446.3 | 6.37 |
| 3 | CUB | Solid concrete floor, automatic circulating scraper (every 3 hours), lagoon emptying every 3-4 month | 3 | 407.9 | 5.43 |
| 4 | CBP | Daily bed tilling, bed emptying up to 6 month. Daily feed alley mechanical cleaning (2 day^-1^) | 1 | 1040 | 10.4 |
| 5 | CBP | Daily bed tilling, bed emptying up to 6 month. Daily feed alley mechanical cleaning (3 day^-1^) | 2 | 6020 | 12.4 |
| 6 | CBP | Daily bed tilling, bed emptying up to 6 month. Daily feed alley mechanical cleaning (1 day ^-1^) | 3 | 2885 | 12.3 |

1. Ramaderia Gonzàlez, 2 La Saireta, 3 Cal Serre, 4 Ramaderia Fontanals, 5 Cal Perches 6 Cal Padrí
