# Supplementary material for: Characterization of microbial populations in two distinct dairy manure management systems: seasonal effect and implications for pollutant gases emissions
Source: J Anim Sci. 2024 Oct 26;102:skae316. doi: 10.1093/jas/skae316 (PMC11641847; doi:10.1093/jas/skae316)
Supplement: skae316_suppl_Supplementary_Table_S2 [file skae316_suppl_supplementary_table_s2.docx]

**Supplementary Material Table 2.** Cows’ performance on both systems under study (CUB and CBP) in summer and winter periods.

| Item | CBP | | CUB | | SEM | *P* value | | |
| --- | --- | --- | --- | --- | --- | --- | --- | --- |
|  | Summer | Winter | Summer | Winter |  | HS^2^ | Se^3^ | HS x Se |
| Cows (nº) | 230 | 273 | 231 | 219 | 50.9 | 0.81 | 0.86 | 0.81 |
| Mean lactation (nº) | 2.2 | 2.2 | 2.3 | 2.3 | 0.049 | 0.07 | 1.00 | 0.52 |
| Parturition interval | 434.6 | 438.6 | 437.6 | 432.0 | 3.89 | 0.82 | 0.92 | 0.55 |
| DMI^1^ (kg day^-1^) | 24.9 | 25.3 | 24.9 | 25.0 | 0.30 | 0.79 | 0.71 | 0.79 |
| Milk yield (kg day^-1^) | 33.5 | 35.1 | 33.6 | 35.1 | 0.45 | 0.91 | 0.12 | 0.91 |

^1^DMI: Dry matter intake; ^2^HS: Housing system; ^3^Se: Season
